# Malaria and Its Toxic Influences—Malarial Hæmaturia

**Published:** 1886-05

**Authors:** W. O’Daniel

**Affiliations:** Bullard’s, Georgia


### MALARIA AND ITS TOXIC INFLUENCES—MALARIAL HÆMATURIA.*

By W. O'DANIEL, A. M., M. D., BULLARD'S, GEORGIA.

What poisonous influences, known to the medical profession of to-day, capable of producing certain specific diseases, are more worthy of profound thought and thorough scientific investigation than those called malarial?

The paucity of literature on a subject so important is indeed remarkable.

Malaria, miasma, spore, cryptogam, germ, bacteria, bacillus malariæ, or whatever else it may be called, frequently causes, in certain localities favorable to its production, both directly and indirectly, a diseased state of the human system upon which supervene maladies fearfully fatal to its unfortunate victims.

Its unchecked ravages are simply disastrous to health and destructive to human life.

It is all-important, then, that the premonitory symptoms of the various forms of malaria should receive much more timely attention than is usual, even by experienced practitioners of medicine, *in order* to prevent the insidious and often unsuspected inroads upon the system, and before the work of degeneration is so far n excess of that of reparation as to weaken the vital powers of life to such an alarming extent as to render recuperation hopeless, even when patients are under the direction and control of medical attendants of unquestioned skill and acknowledged ability.

* Report on the Practice of Medicine from the Sixth Congressional District for 1885.

The uninterrupted and constant prey of this invisible malarial element upon the nervous system and upon the spleen and liver, and indeed upon the whole organism, will inevitably, sooner or later, so sap the constitutions of those within its easy reach as to cause them to succumb to diseases which would otherwise be controlled.

How often have we painfully and helplessly witnessed this sad spectacle despite the best efforts and skill of the most distinguished and learned disciples of Æsculapius.

Many of these troubles could be ameliorated and possibly averted by properly observing and enforcing the laws of hygiene, and adopting *in time* the necessary prophylactic measures, thereby preventing attacks of intermittent, remittent, bilious and pernicious fevers and their usual recurrences.

If preventive means are not employed the systems of those in malarial districts will eventually become so saturated with this morbid element, so dangerous to human health and life, as to finally culminate in the speedy development of what is *now* known by medical men in localities *intensely* malarial as malarial hemorrhagic fever or malarial hæmaturia, a disease which in appearance closely resembles epidemic yellow fever, and would so be diagnosticated by any intelligent physician unacquainted with the causation, etiology and pathology of this comparatively new type of malarial fever, or, if you please, malignant bilious fever, for in our judgment it is nothing more. In its severest and most malignant form it is *almost* a symptom of dissolution. Indeed, in some instances we regard it more as a symptom of disease than a disease *per se*, because it is *always* the result of malarial poisoning and never occurs except in anæmic patients whose blood corpuscles have been disintegrated by malarial complications.

The blood having become impoverished and deficient in some of its normal constituents, and perhaps retaining others that should have been eliminated and thus changed by the absence of some ingredients and the presence of others not properly belonging to it, that it is, *strictly* speaking, no longer blood.

While this life-sustaining fluid is in this abnormal and poisoned condition, caused by neglected, chronic, relapsing intermittents, this peculiar phase of disease develops, and with these surroundings the lives of those attacked are dependent *mainly* upon the severity of the disease. We have seen death ensue, to all appearances, just as a woman dies from post partem hemorrhage, or death from accidental hemorrhage. The impression upon the constitution and upon the circulation is apparently almost identical. Frequently those violently attacked with this fever actually die so suddenly, in a state of congestion and malarial stupor, as to leave no time for human effort to assist nature in bringing on reaction, and thus the end comes *apparently* from sheer exhaustion and for want of blood. Of course there is an ample supply of this abnormal fluid just described, but these toxic changes have rendered it unfit as a life-sustaining and vitalizing fluid. In such cases we are inclined to the opinion that if transfusion ever was indicated, *immediate* recourse to it should be had. We do not desire, however, to be understood as recommending transfusion as a *common* remedy in hemorrhagic fever.

As undisputed evidence to the minds of those in doubt, that the malignancy of malarial fever in certain sections, especially in the cotton growing States, has rapidly increased to an alarming extent within the last two decades, hemorrhagic fever never appeared in Georgia until about 1868.

This statement is corroborated by gentlemen of reliability, eminence and distinction in the medical profession of Georgia, who have seen malarial diseases in all of their different forms and have large experience in their treatment.

Among whom are Doctors Hawkins, of Americus; Bruce, of Bainbridge; Doster, of Blakely; Hilsman, of Albany; Watts, of Montezuma; Smith, of Hawkinsville; Pate, of Sumter county; McHatton, of Macon; Moore, of Macon; and Alexander, of Forsyth.

These gentlemen have very kindly and courteously furnished me with valuable information in regard to malarial hemorrhagic fever, and none of them ever saw a case until 1868, since which time it has made its annual appearance in the localities in which these gentlemen reside, and they agree that enfeebled and broken-down constitutions, from intermittents and their usual recurrences, have nearly always been its victims, and the severity of the attack always, in a degree, commensurate with the intensity of previous exposures and sufferings from malarial influences.

Therefore we contend that this particular type is purely malarial, and that there is a superabundance of malaria, much more than there was twenty years ago, whereby the system becomes surcharged with it, as it were, or, in other words, has more than a sufficiency to produce, or cause, the ordinary chills and fever of former times; hence this virulent form which was unknown before we had this increase of "so-called" malaria.

Now, what is the cause of this overdose or increase of malaria? We answer that, in our opinion, it is because of a want of proper drainage mainly.

In many localities low, damp, marsh and bottom lands were kept thoroughly drained for agricultural purposes before the war between the States; whereas now, for want of efficient labor, the system of drainage by ditching has been, in many instances, totally abandoned, and if not entirely neglected, so imperfectly done that little or no good is derived so far as regards sanitation.

Therefore we believe that the frequent inundations of these lands, in fertile sections where vegetable matter is abundant and undergoing rapid and constant decomposition during the summer and fall months, is the cause of this superabundance and increase of malaria, which we claim is the producing and promoting cause of malarial hæmaturic fever. This may be by some considered hypothetical, but it must be true, for the reason that this fever is only found in localities where drainage is thus neglected, and thereby becomes especially noted for the prevalence of intermittents and other malarial complications.

Much more can be done in the way of preventing this disease than in relieving patients already attacked with it. This is best done by preventing chills and fever. When this is done successfully, we need have no fear of hemorrhagic fever, for it is only the sequel of some of the various forms of malarial complications.

We ought always to reside in localities away from these chillproducing influences, but when this is unavoidable, the malaria must be neutralized by the proper remedies as fast as it is taken into the system. We have known this to be done in many instances by the taking of five grains of quinine on retiring at night, this to be continued, however, from early spring until frost. Should a chill occur, never wait for a second one before commencing treatment.

The antidote is quinine or some of the alkaloids of cinchona. Although quinine is the antidote of malaria, it will not prevent recurrent attacks unless long and judiciously continued. Remedies for the relief of hepatic and splenitic complications should be employed, together with tonic treatment, to insure residents of these fever districts even partial immunity from recurrent attacks of intermittents.

After long experience we have found the following very efficient:

| | | |
|---|---|---|
| ℞. | Quinine sulph. . . . . . | ʒ ij |
| | Ferri sulph. . . . . . . | ʒ iss |
| | Acidi arseniosi . . . . . | |
| | Strychnine aa. . . . . . | gr. ij |

M. Fiat pills No. 60.
Sig. One pill *ter in die* after meals.

| | | |
|---|---|---|
| ℞. | Quinine sulph. . . . . . | ʒ j |
| | Precip. carb. iron . . . . . . | ʒ v |
| | Liq. potass. arseni . . . . | f ʒ iij |
| | Spts. frumenti . . . . . . | |
| | Aqua aa. . . . . . . . | f ℥ viij. |

M. Sig. Dose, tablespoonful *ter in die* after meals.

For engorgement of the spleen and liver, and for torpidity of the liver, we have found proto-iodide of mercury and saccharated calomel, properly administered, among the most efficient remedial agents for this specific purpose. Counter-irritation is often indispensable in the treatment of these vascular organs when in a diseased state.

We have known the spleen and liver, when tender, enlarged and engorged, the result of frequent attacks of intermittents, relieved by blisters when other recognized modes of treatment had utterly failed.

A well-marked chill, or rigor, generally, but not always, develops this disease so plainly by the sudden change of the normal urine to that of a dark, blood-like fluid, and the whole cutaneous surface to a jaundiced appearance, that the diagnosis is unmistakable, even with the unprofessional who are acquainted with it. This remarkable change, so soon after the chill, we suppose to be caused by an occlusion of the ductus communi coledochus, on account of which there is an effusion of bilious matter through the whole system. In violent cases patients frequently lose their mental faculties, remain in a state of partial delirium, with perhaps lucid intervals, until the crisis is over, while the minds of others remain clear all the while. These conditions are dependent entirely upon the virulency of the disease. We have seen forms of the disease so slight as not to confine patients to their beds.

TREATMENT.

One of the first things to consider in the treatment of this disease is to restore the secretions and relieve the hepatic, splenic and gastric congestion.

Saccharated calomel is, perhaps, the best remedy for this, because it is best tolerated by a nauseated stomach. Though by all means avoid excessive purgation.

Promote rest and ameliorate nausea by morphine hypodermically, and beware of too much and too frequent drugging. As the disease is certainly of malarial origin, of course quinine is *one* of our available therapeutic agents. We give it to neutralize malarial poison, to prevent recurrent chills or rigors. We give it to equalize the circulation and lower the temperature; hence the necessity for a sufficiency of quinine to accomplish these ends, *if possible*, and no more.

The idiosyncrasy, temperament, constitution, action of heart, etc., of each individual patient must be carefully considered before the administration of potent remedies, and supportive treatment

*must not* be forgotten. We believe quinine to be a heart-depressant and a nerve irritant, and will increase gastric irritation, which is generally a very annoying symptom, and greatly interferes with successful treatment, because the stomach fails to retain the remedies.

Dilute nitric acid, or sulphuric acid, in ice-water is pleasant and refreshing, and has a decided tendency to check the much-dreaded hemorrhage.

If uremic poisoning is suspected, then it is especially indicated.

If quinine has been taken, its solubility will be rendered certain and its absorption facilitated.

Blistering over epigastrium, with cantharidal collodion, modifies duodenitis and ameliorates gastric irritation. Carbolized emulsion, a mixture of bismuth, carbolic acid and glycerine, is a very efficient remedy in gastric troubles.

Hyposulphite of sodium, in ten or fifteen grain doses, three times daily, assists nature in restoring the kidneys to normal action. Chloride of iron is often necessary in anæmic subjects. Ergot is a valuable remedy *in its place*.

Hemastatics must be employed with caution, as suppression may be induced by their too liberal use, which is a very unfavorable symptom. After convalescence, a generous diet and tonics are advised.

Owing to the seasons last year (1885), the producing causes or malaria were lessened; consequently we had but few *genuine* cases of *malarial* hæmaturia. Only three cases occurred in my practice, although I saw several cases of hæmaturia from other causes, two of which I remember were caused from overdoses of turpentine, and one from traumatism, and a few others from specific causes, which no doubt would have been called *malarial* hæmaturia by a *few* practitioners, anxious to swell their number of cases, and ambitious for unmerited reputations for special skill in the treatment cf this *particular* form of disease.

On the night of January 30th, 1885, I was called to see Willie Lawrence, a child a little over two years old, who had, only a few hours previously to my visit, had a chill, and afterwards passed (so called) bloody urine. Upon examination and inquiry, I

found that the little fellow had for sometime been suffering from recurrent attacks of intermittent fever. He had, for a day or two previous to his chill, been suffering from diarrhœa; therefore we deemed it unwise to interfere with the bowels, as the diarrhœa had been checked. Rest was promoted by small doses of morphine. Quinine given to anticipate an expected paroxysm, and stimulants freely administered to bring about reaction, and aromatic sulphuric acid frequently, in cold water, to dissolve the quinine which had already been taken, and to check the *excessive* hemorrhage. These remedies seemed to have the desired effect and improvement was apparent.

January 31st.—There were symptoms of suppression, when a prescription containing squills, digitalis and spirits of nitre was advised. This, too, had a happy effect, and the urine assumed a normal appearance on the evening of the second day.

Upon this treatment the child was able to sit up in a week, after which time he was put upon a tonic of precipitated carbonate of iron, a little quinine, whisky and water. A good and speedy recovery was the result.

On September 25, 1885, Mr. E. M. Lowe, 26 years old, a man of feeble constitution and a long sufferer from bronchial and pulmonary troubles, and whose residence was a favorable one for malarial diseases, and whose blood had become defibrinated and impoverished to such an extent as to render him a fit subject for malarial hemorrhagic fever, was the unfortunate victim of this malady. His case was of the most violent form.

He died in less than twenty-four hours after his attack, with most of the symptoms of this disease—congestion, gastric irritation, vomiting, copious hemorrhages from the kidneys, and the whole cutaneous surface as yellow as an orange. That the skin should undergo such a remarkable change in so short a time may seem incredible to those unacquainted with the character of the disease, but it is nevertheless true.

Circumstances beyond our control prevented us from being with the patient until just before death.

On November 9th, 1885, we were called to see Mr. Henry Faulk in consultation with Dr. Slappy.

Mr. Faulk was a man about 26 years old, of vigorous constitution, who had always enjoyed perfect health until a few months before this attack. Although he resided in a healthy locality, and one free from miasmatic influences, he contracted chills and fever from frequent exposures in the swamps while hunting during the autumn months. He was sick some days before we visited him. Notwithstanding his former health, vigor and almost unprecedented strength, he came very near succumbing, but after a long and tedious combat with disease, he recovered.

The first case mentioned was the youngest I have ever seen with hemorrhagic fever. So far as my own personal observation goes, the negro is exempt from it. We have known but one mulatto to have the disease. The first case that ever occurred in my practice was in 1871, since which time I have frequently seen it during the fall and winter months.

---

THE TREATMENT OF SERILITY.—The most common cause, according to Hewitt, is a conoidal shape of the cervix. Generally the vaginal portion of the neck is elongated, and the whole cervical canal is bent upon itself. The uterine orifice looks forward; therefore conception is difficult.

Two curative methods are recommended: first, amputation of a portion of the neck; second, division of the cervix by median incision through the posterior wall. A patient having a cervix very much flexed and elongated, suffering with difficult micturition and painful locomotion on account of anteflexion, married four years and having no children, presented herself to Hewitt, who performed the following operation: After drawing down the neck he excised upon the median line of the posterior surface a vertical portion of mucous membrane an inch long and half an inch wide. Then he stitched the edges above and below. The effects of this operation was to shorten the cervix on its posterior wall to draw the os backward and to straighten the vaginal portion of cervix.

When three sutures were inserted the os, which previously looked forward, pointed directly downward. After several days the sutures were removed. The patient subsequently became pregnant.

The author prefers this operation to amputation of the vaginal portion because it preserves the natural os and leaves the cervical canal intact. There is also less mutilation than by that of Emmett's.—*Journal de Medecine, April 4, 1886.*

